# Prefibrotic Myelofibrosis Presenting with Multiple Cerebral Embolic Infarcts and the Rare *MPL* W515S Mutation

**DOI:** 10.1155/2020/8375986

**Published:** 2020-06-19

**Authors:** Stephen E. Langabeer, Lisa Lee Tokar, Laura Kearney, Cathal O'Brien, Kowshika Thavarajah, Aisling Barrett, John McManus, Hilary O'Leary

**Affiliations:** ^1^Cancer Molecular Diagnostics, St. James's Hospital, Dublin D08 W9RT, Ireland; ^2^Department of Medicine for the Elderly, University Hospital Limerick, Limerick V94 F858, Ireland; ^3^Department of Haematology, University Hospital Limerick, Limerick V94 F858, Ireland

## Abstract

Acquired, activating mutations of *MPL* W515 are recognised driver mutations of the myeloproliferative neoplasms (MPN), namely, essential thrombocythemia and primary myelofibrosis. The most common mutation at this codon is W515L with several other mutations also described at a lower frequency. Of these less common mutations, *MPL* W515S has only been reported sporadically with limited information on clinicopathological associations. We describe the case of an elderly man with persistent thrombocytosis presenting with an ischemic cerebral event. Bone marrow biopsy showed evidence of prefibrotic myelofibrosis with targeted sequencing demonstrating the presence of the rare *MPL* W515S mutation. Thrombolytic and cytoreductive therapies resulted in a favorable outcome and follow-up. This case provides additional, necessary, and phenotypic data for the rare MPN-associated *MPL* W515S mutation.

## 1. Introduction

The most commonly acquired driver mutations of the Philadelphia chromosome-negative myeloproliferative neoplasms (MPN) of essential thrombocythemia (ET) and primary myelofibrosis (PMF) are those of *JAK2* V617F, *CALR* exon 9, and *MPL* exon 10. Identification of these mutations constitutes a major diagnostic criterion for disease diagnosis and classification [1]. *MPL* encodes the receptor for thrombopoietin with the first MPN-associated, *MPL* exon 10 mutation described as W515L swiftly followed by reports of several other mutations occurring at this particular tryptophan (Trp) codon in patients with both ET and PMF [2–4]. However, the W515 codon appears to be a hotspot for acquired mutations, and several other somatic and inherited variants of *MPL* (other than in exon 10) have also been annotated in familial and sporadic MPN [5–9].

Morphological comparison of *MPL*-mutated ET with *JAK2* V617F- and *CALR*-mutated ET has shown that *MPL*-mutated ET patients are older than those with other genotypes and have higher peripheral blood platelet counts and bone marrow megakaryocytes than *JAK2* V617F-mutated ET with histological features of prefibrotic myelofibrosis being rare [10]. Review of bone marrow histology of *MPL*-mutated PMF shows decreased vascularity and increased osteosclerosis [11]. Clinically, *MPL*-mutated MPN patients tend to have lower risk of thrombotic events than their *JAK2* V617F counterparts [12–14].

The W515S mutation was first described in *in vitro* as a spontaneously occurring point mutation in the intracellular domain of the protein after transfection of *c-mpl* into Ba/F3 cells that resulted in thrombopoietin-independent, constitutive activation of the receptor [15]. Isolated reports of *MPL* W515S in MPN patients exist in the literature, usually within a cohort, yet limited clinical and pathological associations regarding this specific mutation exist [16–19]. Of these four cases, two patients had annotated clinical histories and in whom no thrombotic episodes were observed.

## 2. Case Report

A 71-year-old man with a persistent high platelet count for four years (range 483–797 × 10^9^/L) presented with dense, right-sided weakness, aphasia, and a decreased level of consciousness. He was known to have hypercholesterolemia for which he was on atorvastatin. He was an ex-smoker for forty years and otherwise did not have any other vascular risk factors. Apart from thrombocytosis, blood count parameters including hemoglobin, hematocrit, white cell count, and white cell differential had always been normal. Initial computerized tomography (CT) scan showed an old, left occipital lobe infarction. CT angiogram did not show any large vessel occlusion. Magnetic resonance imaging scan confirmed multiple, left-sided, and embolic infarcts. Of note, a 72-hour Holter monitor excluded paroxysmal atrial fibrillation as a cause of cerebral ischemia. Standard stroke workup did not identify any other sources of cerebral embolic infarction. He was thrombolysed and subsequently commenced on low-dose aspirin 75 mg daily. He made an excellent recovery, the only residual deficit being that of slightly altered speech.

**Figure 1:**
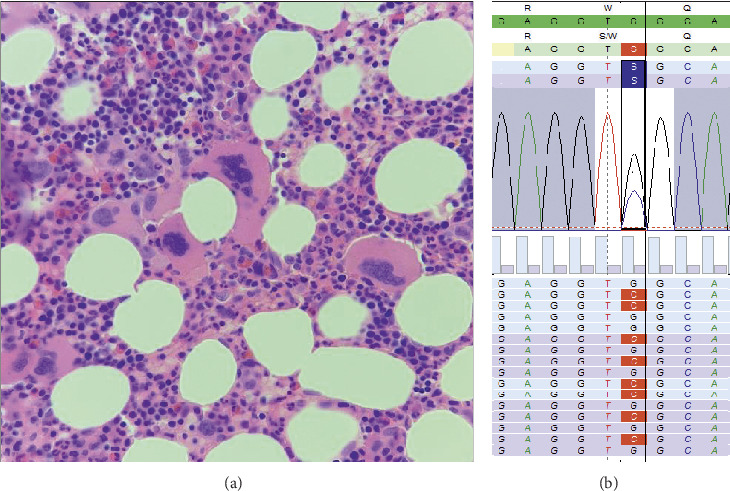
(a) Bone marrow biopsy demonstrating megakaryocyte clustering with nuclear hypolobation and bulbous forms; (b) next-generation sequence analysis (Sequence Pilot, JSI Medical Systems, Ettenheim, Germany) identifying *MPL* p.W515S (c.1544G > C).

Following his discharge from the hospital, he was referred to the hematology service for investigation of the persistent thrombocytosis. Bone marrow aspirate was aparticulate. Bone marrow biopsy was hypercellular with increased numbers of megakaryocytes showing paratrabecular clustering and nuclear hypolobation as well as bulbous forms (Figure 1(a)); granulopoiesis and erythropoiesis looked normal with World Health Organization grade 1 reticulin fibrosis seen that are all features in keeping with a diagnosis of prefibrotic myelofibrosis. An International Prognostic Scoring System score of 1 predicted his median overall survival for more than 10 years [20]. Karyotypic analysis failed on the bone marrow aspirate; however, a next-generation sequencing approach identified p.(Trp515Ser), c.1544G > C mutation (NM_005373.2), usually referred to as *MPL* W515S, at an allele frequency of 35% in the patient's peripheral blood (Figure 1(b)). No *CALR* exon 9 or *JAK2* exon 12 or 14 mutations were identified.

In order to reduce the risk of further thrombotic events in the context of confirmed *MPL* W515S MPN, hydroxyurea 500 mg daily was introduced, resulting in the platelet count normalising within one month of starting this treatment and remaining to date within the normal range. Currently, the patient remains well and is fully capable of carrying out all daily activities. He has a persisting, slight speech impediment and remains on aspirin and hydroxyurea more than twelve months following the ischaemic cerebral event and has not had any bleeding sequelae.

## 3. Discussion

The Trp515 amino acid is located at the transmembrane-cytosolic junction of the MPL receptor controlling dimerization and activation [21]. This codon is recurrently mutated in MPN with the mutations W515L and W515K detected most frequently but with W5151R, W515A, and W515G also described [16, 22], whereas *MPL* W515S has only been sporadically reported in ET and PMF patients [16–19]. In a recent study of 17 active mutants resulting from substitutions at Trp515, W515S resulted in activation of the TpoR/JAK2/STAT5 transcriptional cascade in the absence of the ligand in a similar manner to that of other mutants described in MPN patients thus implicating its oncogenic potential [23].

As MPN are relatively rare, it is probable they represent an underdiagnosed cause of cerebral thrombosis. High clinical awareness is warranted to identify an underlying MPN in order that a prompt treatment is initiated (e.g., antiplatelet, cytoreductive, and phlebotomy) [24, 25]. While some studies have suggested routine screening for *JAK2* V617F in patients presenting with stroke and/or cerebral venous thrombosis (CVT) [26], given the lower risk of thrombosis in *CALR*- and *MPL*-mutated MPN patients and the near absence of these mutations in CVT cohorts [27, 28], wider evaluation of mutation status might be reserved for those patients with sustained abnormal blood count findings.

Detection of MPN-associated mutations is achieved by a number of technological approaches including Sanger sequencing, allele-specific PCR, melt curve analysis, capillary electrophoresis, pyrosequencing, digital PCR, and next-generation sequencing [29]. Given the possibility of rare mutations such as W515S, a diagnostic screening approach covering at least *MPL* exon 10 such as next-generation sequencing is preferable to those techniques that target individually the most prevalent mutations of *MPL* W515L and W515K.

Identification and reporting of such rare *MPL* mutations in conjunction with the clinical aspects in MPN patients allow for correct diagnosis, assessment of any genotype-phenotype relationship, and may provide information on any future therapeutic approaches in similar patients.

## Data Availability

No data were used to support this study.
